# Regulatory efforts to address the access gap for foreign new drugs in China: the priority review program and related policies

**DOI:** 10.1186/s41256-024-00396-5

**Published:** 2025-02-25

**Authors:** Xingyue Zhu, Jinsui Zhang

**Affiliations:** 1 School of Medicine and Health Management, Guizhou Medical University, Guiyang, 550025 Guizhou China; 2 Center of Medicine Economics and Management Research, Guizhou Medical University, Guiyang, China; 3 Department of Health Economics, School of Public Health, Fudan University, Shanghai, China

**Keywords:** Drug lag; launch delay; drug approval; priority review

## Abstract

**Background:**

China has implemented the priority review (PR) program and flexible registration requirements for new drugs with significant clinical value since 2016 to accelerate drug access. We aim to explore the impact of the reform efforts on the drug access gap between China and the US.

**Methods:**

We collected data on the imported new drug approvals that were licensed in China between 2007 and 2023, and measured their launch delays as compared to the US. Difference-in-difference models were used to compare the launch delays of PR approvals and non-PR approvals before and after the implementation of the PR. Propensity score matching was used to construct the imputed PR and non-PR approvals in the pre-PR period.

**Results:**

A total of 410 imported approvals were licensed in China in 2007-2023. Most approvals (316[77.1%]) were licensed after the PR was implemented, of which 189[59.8%] received the PR designation. The difference-in-difference models found that the PR program reduced drug launch delay by 1157.0 days (robust standard error, 571.0; P<0.05) and reduced drug submission delay by 1037.3 days (robust standard error, 520.8; P<0.05). The PR identified drugs with high clinical value and informed flexible registration requirements for them, which accelerated drug submission and market entry.

**Conclusions:**

Our findings proved the importance of value-based prioritization of new drugs and flexibility in the statutory evidentiary standard in the drug approval process. Further efforts from the drug agency are needed to leverage the regulatory flexibility to provide fast market entry of new drugs without compromising their quality.

**Supplementary Information:**

The online version contains supplementary material available at 10.1186/s41256-024-00396-5.

## Background

The access gap for new drugs between countries, also known as the launch delay or drug lag, is a major public health issue in many regions [1–4], which jeopardizes patient health and discourages pharma companies [5]. The drug launch delay was first determined in the US in 1973 [6]. This issue was related to the stringent regulation by the US Food and Drug Administration (FDA) following the thalidomide event and its time-consuming review process [7, 8]. In 1992, the US endorsed the Prescription Drug User Fee Act (PDUFA) to accelerate patient access to new drugs, efforts of which included stipulating the drug review timeframe and creating the priority review pathway to further shorten the timeframe for drugs with high clinical value [9]. Under PDUFA, the FDA’s drug review speed was improved [10], and the launch delay of new drugs in the US was addressed [11]. The US has set an example in terms of bridging the drug access gap, and many jurisdictions are following suit to establish similar expedited review programs. However, the benefits of these programs remain largely unclear.

China was plagued by long-standing launch delay of new drugs as compared to the US and EU [12, 13]. This delay results from two parts, the lengthy review time arising from the redundant review procedure, and the postponed submission of drug applications. Standalone domestic clinical trial in China was the prerequisite to filing a new drug application (NDA) [14]; and after filing, NDAs had to wait an average of 12.3 months to be reviewed due to a severe backlog of applications and understaffing of the Chinese drug agency [15]. The stringent clinical research and development (R&D) requirements and the inefficient review process undermined the interest of foreign drug developers in the Chinese market, and therefore affected their submission decisions. On this account, the Chinese government has rolled out reforms in the drug regulatory system since 2015, which aim to improve access to novel drugs and stimulate innovation [16]. In Feb 2016, the priority review (PR) program was formally launched to provide prior resources for the evaluation of drugs with significant clinical benefits [17]. As the first new review program, the PR has been found to be associated with the faster review speed of the Chinese drug agency and the reduced launch delay of new drugs in China relative to the US, EU, and Japan [18–20]. However, whether the PR program meets its commitment of improving drug access awaits to be answered yet. In this study, we seek to delve into the impacts of the PR program on the launch delay of foreign new drugs in China, in order to understand the implications of the PR and its related policies and help shape future regulatory innovations.

## Methods

### Setting

The scope and benefits of the PR and its related policies were summarized in Table 1. At the time of its creation (2016-2019), the PR offered prior review along with further benefits to expedite drug access: firstly, for drugs against rare conditions, overseas trials could serve as the sole basis for regulatory approval; secondly, for drugs with promising preliminary evidence, conditional approval could be granted before the phase III trial was completed [21]. Thereafter, the exemption of domestic trials and the conditional approval evolved into separate policies [18]; and in 2020, the benefit of the PR was curtailed to a reduction in review time (130 days for PR vs. 200 days for standard review) [22]. It can be seen that the core mechanisms of the original PR have been divided into three separate policies, but which are still closely linked to each other by their common scope. The regulatory incentives we study, which aim to accelerate access to drugs with clinical superiority, are not changed in nature.

**Table 1:** Introduction of the priority review and its related policies

	Priority review		Adoption of overseas trials	Conditional approval
Timeline	2016—2019	2020 and later	2018 and later	2017 and later
Scope	New drugs with high clinical value for the treatment of: (1) cancers; (2) rare conditions; (3) major infectious diseases of HIV, viral hepatitis and tuberculosis; (4) pediatric populations.	New drugs with high clinical value and: (1) addressing urgent medical needs; (2) providing new dosage or new formulation for pediatric populations; (3) treating rare conditions or major infectious diseases; (4) granted with conditional approval; (5) granted with breakthrough therapy.	Drugs with high clinical value for the treatment of: (1) life-threatening diseases; (2) rare conditions; (3) pediatric diseases with unmet medical needs.	New drugs intended to treat serious or life-threatening ailments based on surrogate end points or intermediate results
Benefit	1. communication with the agency before application 2. prior review and evaluation 3. conditional approval based on preliminary evidence for life-threatening diseases 4. exemption of domestic trial for rare conditions	1. communication with the agency before application 2. prior and fast review (130 days)	1. approval based on overseas clinical data, if there is no ethnic sensitivity 2. conditional approval based on overseas clinical data, if ethnic sensitivity exists or remains unknown	1. early approval with the commitment to complete confirmatory trials after marketing 2. priority review

In Feb 2016, the PR was created as a comprehensive program to lead the reform in the Chinese drug approval system. In 2016-2019, the PR provided more benefits than a fast review process: before submitting an application, drug sponsors could apply for communication with NMPA and request a reduction or exemption of domestic clinical trials, or conditional approval based on early-stage evidence. Since 2020, the benefits of flexible registration requirements have been removed from the PR. However, the PR drugs may still apply for conditional approval or exemption of domestic trials, provided that the requisite conditions are met. PR, priority review. NMPA, National Medical Product Administration.

### Design

This retrospective cross-sectional study included the imported drug approvals licensed by the Chinese drug agency, National Medical Product Administration (NMPA), during Jan 1 2007 and Oct 31 2023. All the imported NDAs for new molecular entities and imported biologics license applications (BLA) for new biologics were collected. New indication supplements of prior imported applications were also collected for the post-PR approvals. The US served as our reference country, and hence the approvals that were not licensed in the US were excluded. This sample provided roughly symmetric ‘‘pre’’ and ‘‘post’’ periods around the creation of the PR program.

### Outcome

The main outcome of interest was the launch delay, which could be further divided into the submission delay and the NMPA review time. Launch delay was defined as the gap time between the approval timing of the US FDA and that of China NMPA. Submission delay was measured as the gap time between the submission timing of the FDA and NMPA, reflecting the sponsor’s decision-making that is responsive to the regulatory environment evolution. NMPA review time was defined as the duration from the submission date to the approval date of NMPA, presenting the agency’s performance.

### Data collection

﻿Basic information of each approval was collected from the official NMPA databases [23]: the dates of submission and approval in China, registration class (NDA or BLA), marketing class (initial approval or post-approval supplement), and the approved indications. All the sample approvals were classified into three therapeutic areas in light of their approved indications: cancers, HIV/HCV, and others. Cancers are seriously debilitating conditions with unmet needs, and HIV/HCV are major infectious diseases, which both represent the areas NMPA prioritizes.

 To form the post-PR sample, we used the Listed Drug Database [23] to collect the imported NDAs/BLAs and new indication supplements that were approved between Jan 1 2015 and Oct 31 2023. This period was selected to include pilot PR approvals prior to the formal implementation of the PR. The Listed Drug Database was developed by NMPA in 2016 to disclose drug approval information after NMPA overhauled its system, in which whether an application was designated as PR was disclosed. Consequently, if a drug approval was included in this database and was licensed in 2015 or later, its PR status could be determined, thus assigning it to the post-PR period. However, if an approval was licensed in 2015 or earlier, but not included in the Listed Drug Database, we would be unable to determine its PR status, and it would be assigned to the pre-PR period.﻿ Previous research has shown that global R&D is a strong driver of faster access to new foreign drugs for Chinese patients [24]. Hence, type of the pivotal trial(s) was also determined based on the disclosed drug review reports from the Listed Drug Database. The pivotal trials were categorized into three types: domestic trials, overseas trials, and global trials enrolling sites in China. For approvals supported by more than one pivotal trial, the type of domestic trials would be assigned as long as there was one domestic trial, and the type of global trials would be assigned if there were global trials and overseas trials at the same time.

 To form the pre-PR sample, we used the Chinese Marketed Drug Database of the DrugFuture [25]. This is an unofficial, open database including the drug approvals licensed before 2017, based on which we determined the imported NDAs and BLAs that were approved in China from Jan 1 2007 to Dec 31 2015. Approvals that have been recorded in the Listed Drug Database were excluded. Due to the paucity of available data, post-approval new indications were not gathered for the pre-PR period. The submission dates for the pre-PR approvals were derived from the NMPA’s Application Receipt Database [23], which recorded all the applications submitted to NMPA, including investigational new drugs (INDs) and NDAs/BLAs. Besides, as we found all the pre-PR approvals had filed INDs for domestic clinical trials in China, we assumed they were all supported by domestic studies. Approved indications of the pre-PR approvals were extracted from the labels in YaoZhi business database [26].

 The FDA review and approval information for each approval was likewise used as important variables. Hence,  the information on the FDA’s approval date, submission date, priority review designation, accelerated approval designation, orphan designation, and whether a boxed warning in the approved label was assigned was collected for each approval, using the Drugs@FDA database. Every approval’s FDA review time was also measured, by calculating the duration from the submission date to the approval date in the US. As to the FDA review time, we constructed a dummy variable to roughly reflect the extension of review process. According to the stipulated review timeframe, if the FDA review time exceeded 180 days (6 months) for priority review drugs, or 300 days (10 months) for standard review drugs, we assumed that a review extension was in place. Extended review time may be caused by multiple review cycles that were related to efficacy or safety concerns [27]. It should be noted that, some drugs that were approved by the FDA very early, e.g., before 2000, might have missing information of the submission date in Drugs@FDA. Nonetheless, excluding them from our statistical analysis is not acceptable. These drugs typically have long delays and thus the exclusion of them will lead to bias of estimations. Hence, we established a linear model for the FDA review time, based on which we could fill in the missing values (Table S1-2).

### Statistical analysis

Matching algorithm. We would construct a two-period difference-in-difference (DID) model to evaluate the impacts of the PR program. However, for such a DID model, one major concern was that there were no control group (non-PR approvals) and treatment group (PR approvals) before the PR was implemented. Accordingly, we used one-to-one nearest neighborhood propensity score matching (PSM) to construct the control and treatment groups in the pre-PR period, based on the key features of real non-PR approvals and PR approvals respectively (Supplementary Material A). Rosenbaum bounds were used to assess potential hidden bias in the matching. The Mahalanobis matching with heteroskedasticity-consistent analytical standard errors was used as the sensitivity analysis. The PSM might find some pre-PR approvals were matched to both the real PR approvals and the real non-PR approvals. In the DID models, each of the dual-matched approvals would be randomly assigned to either the imputed PR group or the imputed non-PR group, and we would then conduct 1000 replications of the random assignment to produce robust estimations of the treatment effects.

Difference-in-difference specification. Our DID model took the following formula:

1 $$\begin{array}{*{20}c} {L_{n} = \beta PR_{n} \times Post_{n} + PR_{n} + Post_{n} + {\varvec{u}}_{n} + \varepsilon_{n} } \\ \end{array}$$

Here, ${L}_{n}$ measured the length of launch delay of a specific approval $n$. ${PR}_{n}$ indicated whether the approval $n$ was in the treatment group, which took on a value of 1 if $n$ actually received the PR designation or was matched to the real PR approvals. ${Post}_{n}$ indicated whether the approval $n$ fell within the post-treatment period, which took on a value of 1 if $n$ was registered in the Listed Drug Database. ${PR}_{n}\times {Post}_{n}$ indicated the interaction of treatment and period, and the coefficient $\beta$ would estimate the treatment effect of the PR. ${{\varvec{u}}}_{n}$ was a vector of controls, including the FDA’s designations of priority review, accelerated approval and orphan drug, the FDA’s boxed warning at approval, the FDA review extension, the therapeutic areas (cancers, major infectious diseases, or others), the type of the pivotal trial enabling the NMPA approval (domestic trials, overseas trials, or global trials with sites in China), the approval class (NDA or BLA), the marketing class (initial marketing approval or new indication supplement), and the year of the NMPA approval. The DID models for the submission delay and the NMPA review time were defined in the same way.

Event studies. We examined the parallel trends assumption by estimating the following equation:

2 $$\begin{array}{*{20}c} {L_{n} = \mathop \sum \limits_{t \ne z} \rho_{t} PR_{n}^{\prime } \times Post_{tn} + PR_{n}^{\prime } + Post_{tn} + {\varvec{u}}_{n} + \nu_{n} } \\ \end{array}$$

Where ${Post}_{t}$ was an indicator for each year from 2007 to 2023, indicating the fictitious PR timing, and ${\rho }_{t}$ was the coefficient of interest. $z$ was 2015 which was forced to be zero to avoid perfect collinearity. For $t\in [\text{2007,2014}]$, statistically insignificant $\widehat{{\rho }_{t}}$ would provide certain evidence in favor of the parallel trend assumption. Noted that, ${PR}_{n}{\prime}$ indicated whether the approval $n$ was in the treatment group; and here we used three definitions of the pre-PR treatment and control groups to probe the sensitivity of pre-trends tests. First, we only considered the exclusively matched PR (${PR}_{n}{\prime}=1$) and non-PR approvals (${PR}_{n}{\prime}=0$) as the pre-PR sample. Secondly, the exclusively matched PR approvals were considered as pre-PR treatment group (${PR}_{n}{\prime}=1$), while the exclusively matched non-PR and the dual-matched approvals were deemed as pre-PR control group (${PR}_{n}{\prime}=0$). Thirdly, the exclusively matched PR and the dual-matched approvals were assigned the value of 1 for ${PR}_{n}{\prime}$, while the exclusively matched non-PR approvals were assigned the value of 0.

Mechanism analysis. To investigate the mechanism, we analyzed the different effects of the PR among orphan and non-orphan drugs. NMPA has not established orphan designation yet. Thus, we used the FDA’s orphan designation as the identifier instead. Besides, the adoption of overseas trials and the NMPA conditional approval program are also factors related to the PR. We examined the mediation effects of pivotal trial type and conditional approval program on the relationship between the PR and the drug delay (Supplementary Material B). Due to that the conditional approval and the adoption of overseas trial were first proposed with the PR in 2016, the mediation analysis only covered the post-PR approvals. The significance level was set to be 0.05 for 2-tailed tests, and robust standard errors were reported. Stata version 15 (StataCorp LP) was used to perform the analysis.

## Results

### Summary characteristics

**Fig. 1:**
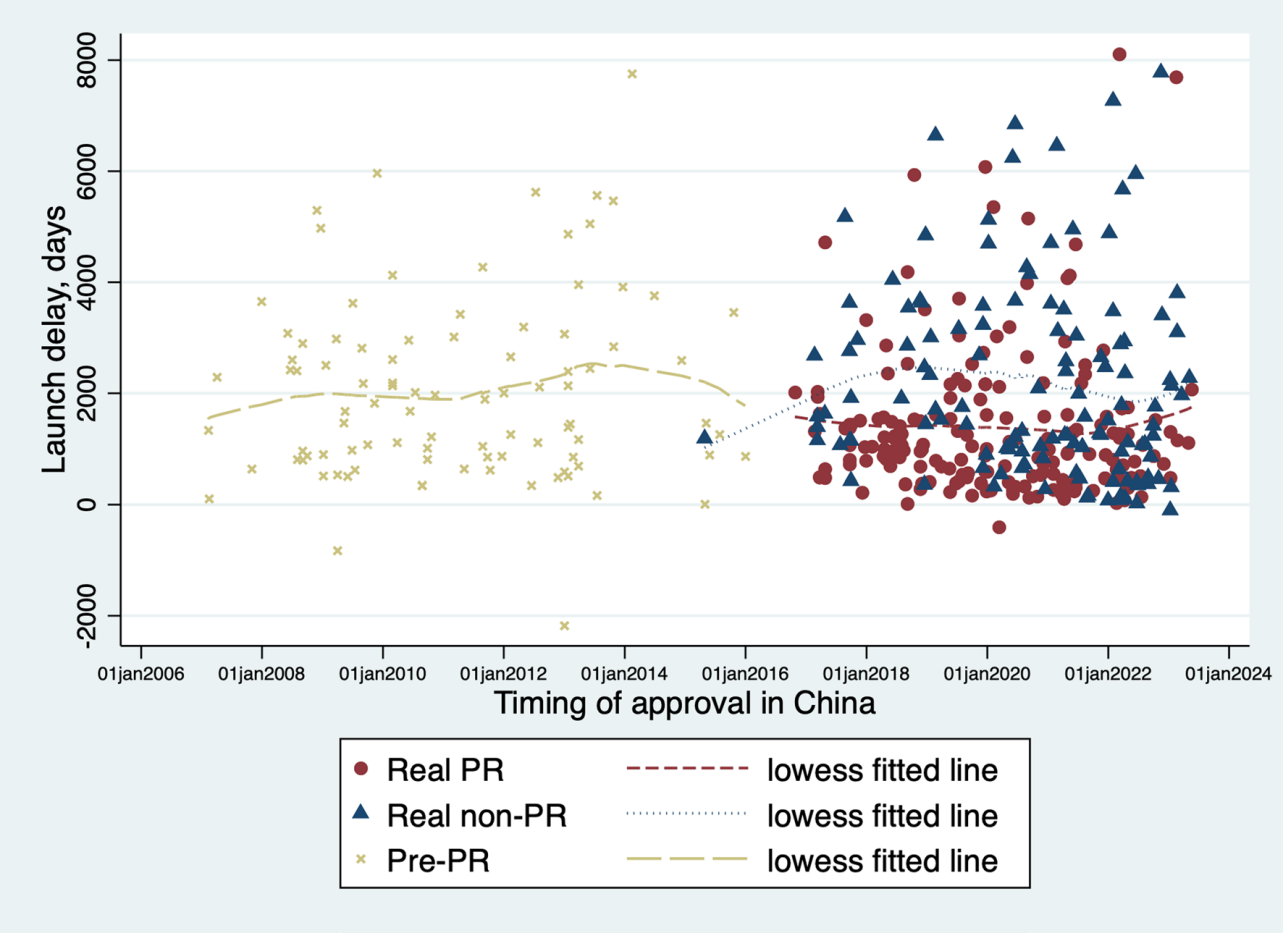
Distribution of drug launch delay, in terms of the PR status. Notes: Red circles denoted the approvals receiving the PR designation after the PR was implemented (real PR). Blue triangles denoted the approvals without the PR designation after the PR was implemented (real non-PR). Yellow x denoted the approvals licensed before the PR was implemented (pre-PR). The fitted dash lines were constructed by the LOWESS (locally weighted scatterplot smoothing) method, which indicated the tendencies of the launch delay. The pre-RP approvals and the real non-PR approvals had similar trends, but the PR approvals presented shorter launch delay. PR, priority review

A total of 410 imported approvals were authorized by NMPA between Jan 1 2007 and Oct 31 2023 (Table 2). Most approvals (316[77.1%]) were licensed by NMPA after the PR program was initiated, of which 189(59.8%) received the PR designation. The non-PR approvals accounted for 53.9% of the total, containing both real non-PR and pre-PR approvals. Some key features showed discrepancies between the PR approvals and non-PR approvals. The PR approvals tended to have more designations of the FDA’s priority review, orphan drug, and accelerated approval, and were more likely to be approved based on foreign trials or global trials including Chinese data. The length of launch delay, submission delay, and the NMPA review duration also differed between the PR approvals and the non-PR approvals. The time trend of launch delay was depicted in Figure 1, presenting an overall trend of stabilization for the pre-PR approvals.

**Table 2:** Characteristics of unmatched sample. Values are counts (percentages).

Variable	PR approval^a^	Non-PR approval^b^	P value
N=189	N=221		
Approval class			
Initial approval	137(72.5%)	155(79.1%)	
New indication supplement	52(27.5%)	66(29.9%)	0.600^c^
Registration class			
NDA	116(61.4%)	148(67.0%)	
BLA	73(38.6%)	73(33.0%)	0.238^c^
FDA priority review			
Yes	151(79.9%)	99(44.8%)	
No	38(20.1%)	122(55.2%)	<0.01***^c^
FDA orphan designation			
Yes	102(54.0%)	59(26.7%)	
No	87(46.0%)	162(73.3%)	<0.01***^c^
FDA accelerated approval			
Yes	39(20.6%)	19(8.6%)	
No	150(72.4%)	202(91.4%)	<0.01***^c^
FDA boxed warning at approval	0.3(0.03)	0.3(0.03)	
Yes	52(27.5%)	69(31.2%)	
No	137(72.5%)	152(68.8%)	0.412^c^
Pivotal trial type			
Domestical trial	41(21.7%)	136(61.5%)	
Overseas trial	69(36.5%)	35(15.8%)	
Global trial enrolling China-based sites	79(41.8%)	50(22.6%)	<0.01***^c^
Therapeutic area			
Cancers	93(49.2%)	61(27.6%)	
HIV/HCV	16(8.5%)	13(5.9%)	
Others	80(42.3%)	147(66.5%)	<0.01***^c^
FDA review times, days, mean(SD)	278.7(20.3)	369.5(17.6)	<0.01***^d^
Launch delay, days, mean(SD)	1385.7(96.8)	2118.8(115.3)	<0.01***^d^
Submission delay, days, mean(SD)	1284.7(99.3)	1898.6(112.7)	<0.01***^d^
NMPA review time, days, mean(SD)	379.7(13.4)	583.1(21.4)	<0.01***^d^

^a^ PR approvals indicated the approvals receiving the PR designation after the PR was implemented. ^b^ Non-PR approvals included the approvals without the PR designation after the PR, and all the approvals licensed before the PR was implemented. ^c^ P values were from chi-square test. ^d^ P values were from independent t-test. PR, priority review. NDA, new drug application. BLA, biologics license application. NMPA, National Medical Product Administration. SD, standard deviation. *p < 0.10 **p < 0.05 ***p < 0.01.

The PSM resulted in 20 approvals exclusively matched to the real PR approvals, and 16 approvals exclusively matched to the real non-PR approvals, which were respectively used as the imputed treatment and control groups for the pre-PR period (Table S3). There were 30 approvals that matched both the real PR and non-PR approvals. Balance tests were displayed in Table S4. In the matched sample, features between the treatment and control groups differed significantly (Table S5), which is due to that the PR approvals are inherently distinct from the non-PR approvals. Based on the matching, the PR program was found to relate to shorter launch delay (mean: 1472.4 days vs 2162.1 days, P<0.01). Rosenbaum bounds showed that the matching for PR approvals was sensitive to moderate hidden bias (Table S6).

### Impacts of the PR program

Table 3 shows the impacts of the PR program on the length of launch delay. A reduction of 1157.0 days (3.2 years) in the launch delay was attributed to the PR program. One thousand replications were further performed with the 30 dual-matched approvals randomly allocated into the imputed PR or non-PR, the results of which remained robust (Figure S1): 73.2% of the estimations revealed the significant impacts of the PR on the launch delay, and the mean reduction of launch delay was 949.8 days (SD, 233.4). The PR program also considerably shortened the submission delay by 1037.3 days, but exerted statistically insignificant impact on the review process of NMPA. The robust check largely supported the PR’s benefit on the new drug submission delay. In most estimations (66.3%), the PR was able to bring about a significant reduction of the submission delay, with a mean reduction of 842.4 days (SD, 211.5). However, the results for the NMPA review time basically remained insignificant (Figure S2-S3). The event studies did not find evidence about the pre-trends (Figure S4-S6). Moreover, it was found that the PR’s effects were significant at the early stages of its implementation (2016-2018), and tended to attenuate later (2019-2023). This may be related to the continuous decline of drug delays of non-PR approvals since 2019 (Figure S7).

**Table 3:** Impacts of the PR on drug delays.

Variable	Launch delay		Review time		Submission delay	
	Without covariates	With covariates	Without covariates	With covariates	Without covariates	With covariates
PR	157.5 (587.5)	513.3 (548.0)	13.3 (133.8)	75.7 (137.5)	84.4 (552.0)	499.5 (499.6)
PR $\times$ Post-PR	−937.3 (615.7)	−1157.0** (571.0)	−130.9 (136.4)	−175.0 (139.8)	−795.9 (582.6)	−1037.3** (520.8)
Constant	2135.2*** (452.8)	6195.9 (88704.2)	768.7*** (90.4)	24280.7 (17645.5)	1887.1*** (394.1)	10836.8 (85327.1)
N	352	352	352	352	352	352
Adj-${R}^{2}$	0.0625	0.2816	0.1947	0.3373	0.0483	0.2824

Observations incorporated the post-PR approvals and the pre-PR approvals that were exclusively matched to either the real PR or the real non-PR. Robust standard errors were in parenthesis. Controls included were the FDA’s designations of priority review, accelerated approval and orphan drug, the FDA’s boxed warning at approval, the FDA review extension, the therapeutic areas [cancers, major infectious diseases (HIV/HCV), or others], the type of the pivotal trial enabling the NMPA approval (domestic trial, overseas trial, or global trial with sites in China), the approval class (NDA or BLA), the marketing class (initial marketing approval or new indication supplement), and the year of the NMPA approval. PR, priority review. NMPA, National Medical Product Administration. NDA, new drug application. BLA, biologics license application. *p < 0.10 **p < 0.05 ***p < 0.01.

### Mechanism analysis

Table 4 displays the different impacts of the PR across orphan and non-orphan approvals. The submission gap for orphan approvals significantly declined by 2515.4 days; while for non-orphan approvals, such an impact was not detectable. The findings were robust after the dual-matched approvals were introduced (Figure S8-S9).

**Table 4:** Impacts of the PR on the delays among orphan and non-orphan approvals^a^.

Variable	Review time		Submission delay	
	Orphan	Non-orphan	Orphan	Non-orphan
PR	227.8 (153.3)	22.8 (182.2)	1698.6 (1045.6)	155.5 (523.2)
PR $\times$ Post-PR	−271.9* (163.1)	−153.6 (183.5)	−2515.4** (1107.0)	−378.0 (562.7)
Controls	Y	Y	Y	Y
N	151	201	151	201
Adj-${R}^{2}$	0.2838	0.3729	0.3180	0.3572

^a^Orphan approvals were defined as the approvals receiving the FDA’s orphan designation. Observations incorporated the post-PR approvals and the pre-PR approvals that were exclusively matched to either the real PR or the real non-PR. Robust standard errors were in parenthesis. Controls included were the FDA’s designations of priority review, accelerated approval, the FDA’s boxed warning at approval, the FDA review extension, the therapeutic areas [cancers, major infectious diseases (HIV/HCV), or others], the type of the pivotal trial enabling the NMPA approval (domestic trial, overseas trial, or global trial with sites in China), the approval class (NDA or BLA), the marketing class (initial marketing approval or new indication supplement), and the year of the NMPA approval. PR, priority review. NMPA, National Medical Product Administration. NDA, new drug application. BLA, biologics license application. *p < 0.10 **p < 0.05 ***p < 0.01.

The mediation effect analysis showed the type of pivotal trials and the conditional approval were both complementary mediators of the PR program (Table S7). The mediation effect of trial type was statistically significant with 34.6% of the total effect of the PR program on submission delay being mediated; while the mediation effect of the conditional approval significantly accounted for 22.9% of the total effect. Nearly 60% of the effect of the PR program was mediated.

### Robustness

We performed several robustness checks. Firstly, the Mahalanobis matching was used as the alternative matching method. It generated more imputed non-PR approvals but less imputed PR approvals (Table S8), based on which the results of DID models were similar to the above main results (Table S9).

Secondly, the creation of PR may also encourage the submission of non-PR drugs by enhancing the confidence of drug firms in the Chinese market. Such spillover effects can discount the estimated magnitude of the PR’s effects. Accordingly, we investigated the effects of the PR’s implementation on the delays of non-PR approvals, and it showed that the spillover effects were not significant (Table S10). This can help us alleviate the concern about potential spillover effects.

Thirdly, the imbalanced inclusion of indication supplements for pre- and post-PR periods might raise concerns about the robustness of our results. The indication supplements were included to enlarge the sample and facilitate the matching algorithm, but they were not collected for pre-PR period due to the paucity of open data. If supplements were excluded, the real non-PR approvals would suffer a heavy sample loss (more than half), based on which the matching results were poor and unable to support the subsequent analysis. However, the imbalanced inclusion of supplements might introduce some confoundings if the effects of PR varied between initial approvals and indication supplements. An indication supplement may require less development cost and have higher regulatory success rate as the drug’s safety and efficacy profiles have been understood to some extent. As such, the delays of supplements may differ from initial approvals. Although it was not feasible to collect indication supplements for the pre-PR period using open data, we can explore the influence of including supplements only in the post-PR period. We analyzed the effects of registration class on drug delays as well as its interaction with the PR designation, using the post-PR sample (Table S11). It showed that indication supplements had a shorter launch delay, which was mainly attributed to their shorter review process; but the submission delay was not affected significantly. For launch delay and submission delay, given that the effects of PR didn’t differ across initial approvals and indication supplements, the inclusion of supplements had a limited influence. For review time, given that the effects of PR were larger in initial approvals than in supplements, our sample with the inclusion of supplements only in the post-PR period might underestimate the true effect of the PR.

## Discussion

The PR program is created to prioritize resources on the drugs with high clinical interest, reflecting NMPA’s concern on drug benefits and its openness toward adaptive and flexible requirements for drug market authorization. We demonstrate that, the PR program combined with other policies related to clinical benefits is effective to narrow the drug accessibility gap between China and the US. The rationale of the PR program is prioritizing drugs in accordance with the magnitude of clinical benefit and enforcing differentiated review policies for different drugs; but the core of PR is more to identify the new drugs with salient clinical improvements than to shorten the review timeline. Such identified drugs have their values recognized and then, are promising to receive other favorable policies that allow flexible deliberations and decisions on a case-by-case basis. When the anticipated benefits of a drug qualify it for the PR, its additional trials in China may be exempted or conducted after marketing, even with uncertainties in its efficacy in the Chinese population. This can incentivize the drug innovators home and abroad to make value-oriented investments and to submit the marketing applications of drugs with high therapeutic value as quickly as possible. For foreign products, the early drug submission and approval in China can facilitate longer patent life after marketing and bring higher returns. The PR program and its related policies indicated an improvement in the Chinese regulatory environment, according to which pharma companies could expect better performance and adopted a more proactive drug launching strategy. Rare diseases are severely afflicted with under-treatment in China [28, 29], which makes orphan drugs urgently needed and thus significantly responsive to the regulatory incentive. It is noteworthy that after several years of the PR’s creation, its positive effects on drug delays began to attenuate, which may be related to the effects of other initiatives on non-PR drugs. Since China joined in International Conference on Harmonization in 2017, NMPA has allowed concurrent phase I multi-regional trials in China, and allowed global trials enrolling China-based sites as the drug approval basis [14]. Also in 2017, NMPA changed the approval system for domestic clinical research facilities into a filing system to increase the clinical trial capacity in China [30]. As compared to the PR and its related policies that focus on drugs with clinical superiority in seriously debilitating diseases, the clinical trial regulation reforms benefit all medications, which can promote China's participation in the global drug co-development for common diseases with its large patient pool and quick enrolment, and enable fast drug submission. However, the Chinese R&D environment for rare conditions remains to be improved, as lack of epidemiological data, under-diagnosis, mis-diagnosis and insufficient infrastructure are prevalent [31, 32], which undermine global co-development and preclude faster drug access. Besides, China strengthened its regulations on human genetic resources in 2019, which mandates stricter control on the human genetic resource materials in clinical research [33]. Given the fundamental importance of clinical genomics in cancers and rare conditions, international drug developers face more hurdles in integrating China into their global trials, particularly early-stage trials, for these serious diseases [34]. Hence, non-PR drugs may benefit more from the clinical trial regulation reforms than PR drugs. To further address the drug lag issue, more regulatory efforts on drug development and review for urgently needed drugs, such as synchronized development mechanism and concurrent review system across regions, can be valuable.

We found less impact of the PR on the review time. As the program directly acting on the review process, the PR was assumed to have some influence. One potential reason is that, NMPA required sponsors to self-examine the clinical trials of marketing applications in 2015 and then launched the authority’s inspection in 2017 to crack down on data fraud [35]. The inspections would lead to the extension of the review process, and even withdrawal of applications. However, due to the limitations of open-source data, we are unable to identify the affected drugs and to determine the duration of inspections. Our results may underestimate the impact of the PR on the review time.

This study highlights the importance of providing priorities and flexibilities to clinically meaningful novel technologies. In the conditional approval and its analogue expedited programs used by other regulators [36], surrogate endpoints and single-arm trial design serve as the basis for licensure in replace of survival improvements demonstrated by randomized trials, which brings greater flexibility in the regulatory decision-making for drug approval. However, the imperfect correlation between surrogate benefits and overall survival has raised concerns that the involved drugs may fail to prove effective [37, 38]. The well-defined criteria for surrogacy validity, the refined methodology to assess a product’s clinical benefits at early stages, and the comprehensive risk management throughout the product lifecycle are beneficial to ensure these programs ultimately deliver on their promise of improving access to high-value drugs.

Drug agencies need adequate resources to fulfill their commitments of conducting a timely and prudent review process. Further regulatory innovations providing more priorities and flexibilities for certain drugs or diseases likewise need a sizable investment of resources by drug agencies, such as novel review programs requiring in-depth engagement of the agencies with the sponsors, new guidance for industry, post-marketing surveillance on drug safety events and continuous monitoring of drug benefit verification, and government funding to finance and guide the scientific research and spur innovation in specific diseases that are deemed of the top priority in clinical need and public health impact. One important reason that PDUFA has been successful in accelerating drug review speed and drug access is that, it mandates substantial user fees from drug companies to support the fast drug review process and the related infrastructure [39]. At present, the review fees for imported drugs levied by NMPA are 593,900 RMB per strength ($83,325 or €76,285, using Dec 2023 exchange rates), which is considerably lower than the FDA ($4,048,695 in fiscal year 2024) [40] and the European Medicine Agency (€345,800 per strength) [41]. Moreover, NMPA’s charge is fixed, while the FDA and the EMA routinely adjust their application fees. Since the public funding is constrained, NMPA should consider dynamic review fees, which are adjusted according to the operational costs, the need of hiring and retention of professionals, the requirements of strategic planning, and the inflation.

### Limitations

Firstly, we selected several factors related to the drug benefit profiles to produce pre-PR treatment and control groups, but which might not be completely reflective of the qualifications of the PR program, as indicated by the Rosenbaum bounds. Future research can consider a matching algorithm based on the magnitude of clinical benefit of each drug, which is directly related to the PR policy. Secondly, the used pre-PR samples were relatively small, particularly after matching, which may influence the robustness of results. To include indication supplements for the pre-PR period using commercial databases may address this concern. Thirdly, we used the FDA’s orphan designation to define orphan drugs in the mechanism analysis, as NMPA and the health sector in China do not clarify the definitions of rare diseases and orphan drugs. Nevertheless, the scope of the FDA’s orphan designation may not be entirely applicable to China. Fourthly, our study covered the period when the Chinese regulations fast evolved, and there are other policies that may affect drug access not involved in this work. The effects of the reforms related to clinical trials need to be investigated with more sufficient data in the future. Lastly, the impacts of the PR program should be cautious to be extrapolated to other contexts where an analogous expedited program is carried out. The mechanism analysis shows that, the PR plays its role in shortening the access gap largely by combining with other strategies, in which the PR serves as a key indicator of clinical value and informs other favorable policies. This suggests the importance of clinical benefit-oriented regulations across the pharmaceutical R&D and regulatory review process. The PR’s impacts are unclear when used with different policy portfolios. But to accelerate access to new drugs, the priority review, as well as the value-oriented regulations acting on the R&D process, should be integrated into a country’s regulatory framework.

## Conclusions

The PR program and the flexible registration requirements prove NMPA’s intensive supports for drugs with clinical significance, thereby attracting drug submission and reducing the access gap for new drugs between China and the US. Orphan drugs benefited more from the PR program and its related policies. The findings confirm the effectiveness of the value-based prioritization of new drugs and the regulatory flexibilities in the drug approval process. More efforts from the drug agency are needed to refine the value-oriented drug regulations to ensure timely and safe drug access.

## Supplementary Information

Additional file1 (DOCX 3682 KB)

## Data Availability

All data are publicly available.
