# Supplementary material for: Regulatory efforts to address the access gap for foreign new drugs in China: the priority review program and related policies
Source: Glob Health Res Policy. 2025 Feb 25;10:7. doi: 10.1186/s41256-024-00396-5 (PMC11853587; doi:10.1186/s41256-024-00396-5)
Supplement: Supplementary file 1 — Additional file1 (DOCX 3682 KB) [file 41256_2024_396_MOESM1_ESM.docx]

Supplementary Material

[Supplementary Material A: Matching Algorithm 3](#_Toc171253675)

[Supplementary Material B: Mediation Model Specification 4](#_Toc171253676)

[Supplementary Material C: Supplemental tables and figures 5](#_Toc171253677)

[Table S1. Linear regression on the FDA review times. 5](#_Toc171253678)

[Table S2. Filling the missing values of the FDA review times. 5](#_Toc171253679)

[Table S3. Counts of the treatment and control groups. 5](#_Toc171253680)

[Table S4. Balancing statistics. 6](#_Toc171253681)

[Table S5. Characteristics of the matched sample. Values are counts (percentages). 7](#_Toc171253682)

[Table S6. Sensitivity analysis for the one-tailed P-value for testing the null hypothesis of no treatment effect of treatment periods on drug delays length in post-PR approvals and pre-PR approvals matched for the FDA approval characteristics ^a^. 8](#_Toc171253683)

[Figure S1. Robustness for the impact on launch delay: 1000 regressions with random assignment of the dual-matched approvals. 9](#_Toc171253684)

[Figure S2. Robustness for the impact on NMPA review times: 1000 regressions with random assignment of the dual-matched approvals. 10](#_Toc171253685)

[Figure S3. Robustness for the impact on submission delay: 1000 regressions with random assignment of the dual-matched approvals. 10](#_Toc171253686)

[Figure S4. Drug delays event studies: the exclusively matched PR and non-PR approvals were used as the pre-PR samples. 11](#_Toc171253687)

[Figure S5. Drug delays event studies: the exclusively matched PR approvals were used as imputed PR approvals, while the exclusively approvals matched non-PR and the dual-matched approvals were used as imputed non-PR approvals. 12](#_Toc171253688)

[Figure S6. Drug delays event studies: the exclusively matched PR approvals and the dual-matched approvals were used as imputed PR approvals, while the exclusively matched non-PR approvals were used as imputed non-PR approvals. 12](#_Toc171253689)

[Figure S7. Trends of drug delays in the post-PR period. 13](#_Toc171253690)

[Figure S8. Robustness for the impact on submission delay for orphan drugs: 1000 regressions with random assignment of the dual-matched approvals. 14](#_Toc171253691)

[Figure S9. Robustness for the impact on submission delay for non-orphan drugs: 1000 random assignments of the dual-matched approvals. 14](#_Toc171253692)

[Table S7. Mediation analysis results. 15](#_Toc171253693)

[Table S8. Results of Mahalanobis matching. 15](#_Toc171253694)

[Table S9. Impacts of the PR on drug delays with Mahalanobis matching. 16](#_Toc171253695)

[Table S10. Effects of implementing PR among non-PR approvals. 17](#_Toc171253696)

[Table S11. Effects of registration class on drug delays 17](#_Toc171253697)

**Supplementary Material A: Matching Algorithm**

We used one-to-one nearest neighborhood propensity score matching (PSM) to construct the control and treatment groups in the pre-PR period, based on the key features of real non-PR drugs and PR drugs respectively. In that the PR program emphasizes the clinical benefits of a drug, we employed the following variables as proxies for a drug’s clinical benefits: the FDA’s priority review designation, accelerated approval designation, orphan designation, boxed warning in the approved label, and the review times. The FDA priority review favors drugs with superior clinical benefits over known therapies, the FDA accelerated approval is designed for life-threatening conditions, and the FDA orphan designation indicates rare diseases. The boxed warning issued by the FDA presents significant safety issues, which relates to the risk-benefit profiles of a drug. Existing findings on the relationship between the review times of an agency and a drug’s risk-benefit profiles are mixed ^[[1]](#footnote-1),^^[[2]](#footnote-2),^^[[3]](#footnote-3)^. However, we used the FDA review times, along with the aforementioned four variables, as the covariates for matching. The FDA review times was incorporated to generate continuous propensity scores, since the other four variables were all binary and they would yield a limited number of propensity scores with which only drugs with exactly the same score could be matched.

**Supplementary Material B:** **Mediation Model Specification**

To explore the channels behind the effects of the PR, we consider two factors that are correlated with the PR: the adoption of overseas clinical data and conditional approval. Before 2020, both the policies were benefits of the PR. After 2020, the two policies are also related to the PR in terms of their scopes. First, the adoption of evidence from overseas trials is itself a sign that the drugs treat serious conditions with unmet needs, making it very likely for them to be granted with the PR. Second, NMPA’s conditional program is established to grant early approvals based on surrogate endpoints for promising drugs against life-threatening diseases. A surrogate endpoint is a marker - a laboratory measurement, radiographic image, physical sign or other measure that is thought to predict clinical benefit, but is not itself a measure of clinical benefit. The conditional approval can be granted to imported drugs with uninvestigated ethnic sensitivity, as well. Drugs that are conditionally approved are mandated to conduct post-approval studies to verify the clinical benefits, or to clarify the ethnic sensitivity in the Chinese population. After the conditional approval became a sperate expedited program in 2020, it is stipulated that conditional approvals can be granted the PR simultaneously. Both the acceptance of overseas trials and the conditional approval program are important policies facilitating fast market entry of drugs with clinical significance. Preliminary analysis of chi-square test found the PR program was significantly associated with drug approvals based on foreign clinical data (P=0.025) and with NMPA’s conditional approval program (P<0.001).

As such, we conducted the mediation analysis to explore potential mechanisms of the PR program by specifying:

$${subL}_{i}=\beta_{c}{PR}_{i}+\boldsymbol{w}_{i}+\varepsilon_{i1}$$

$${Condition}_{i}=\beta_{a_{1}}{PR}_{i}+{Trial}_{i}+\boldsymbol{w}_{i}+\varepsilon_{i2}$$

$${Trial}_{i}=\beta_{a_{2}}{PR}_{i}+{Condition}_{i}+\boldsymbol{w}_{i}+\varepsilon_{i3}$$

$${subL}_{i}=\beta_{c^{'}}{PR}_{i}+{\beta_{b_{1}}Condition}_{i}+{\beta_{b_{2}}Trial}_{i}+\boldsymbol{w}_{i}+\varepsilon_{i4}$$

The established models examined the mediation effects of NMPA’s conditional approval program, and the type of the NMPA’s pivotal trial. The analysis only involved the post-PR approvals. ${subL}_{i}$ was the submission delay for drug $i$, and ${PR}_{i}$ indicated whether $i$ received the PR designation. ${Condition}_{i}$ took on a value of 1 if $i$ received the conditional approval designation, otherwise a value of 0. ${Trial}_{i}$ indicated the trial type, which took on a value of 0 if $i$ had domestic trial as the pivotal trial, 1 if $i$ had overseas trial as the pivotal trial, and 2 if $i$ was supported by global trials with China-based sites. $\boldsymbol{w}_{i}$ was a vector of controls, including the FDA’s designations of priority review, accelerated approval and orphan drug, the FDA’s boxed warning at approval, the FDA review extension, the therapeutic areas (cancers, major infectious diseases, or others), the approval class (NDA or BLA), the marketing class (initial marketing approval or new indication supplement), and the year of the NMPA approval.

**Supplementary Material C: Supplemental tables and figures**

**Table S1. Linear regression on the FDA review times.**

| Variable | Coefficient | Robust standard error | P value |
| --- | --- | --- | --- |
| NDA | 22.9 | 26.1 | 0.364 |
| FDA priority review | -177.0 | 33.9 | <0.001*** |
| FDA orphan designation | 4.4 | 23.6 | 0.854 |
| FDA accelerated approval | 12.7 | 25.7 | 0.623 |
| FDA boxed warning at approval | -9.2 | 23.7 | 0.699 |
| Therapeutic area: cancers |  |  |  |
| HIV/HCV | 36.4 | 21.7 | 0.095* |
| Others | 140.9 | 27.9 | <0.001*** |
| Constant | 316.8 | 60.8 | <0.001*** |
| N |  |  | 406 |
| Adj-$R^{2}$ |  |  | 0.2192 |

Notes: NDA, new drug application. HIV, human immunodeficiency virus. HCV, hepatitis C virus. *p < 0.10 **p < 0.05 ***p < 0.01.

**Table S2. Filling the missing values of the FDA review times.**

| Drug | FDA approval date | FDA priority review | Therapeutic area | Imputed review times of the FDA, days |
| --- | --- | --- | --- | --- |
| Omalizumab | 20jun2003 | No | Others | 457.6647 |
| Gadoteridol | 16nov1992 | Yes | Others | 280.6617 |
| Imiglucerase | 23may1994 | Yes | Others | 280.6617 |
| ADVATE | 25jul2003 | No | Others | 457.6647 |

**Table S3. Counts of the treatment and control groups.**

|  | Total | Non-PR | PR | Dual-matched | Unmatched |
| --- | --- | --- | --- | --- | --- |
| Pre-PR | 94 | 16 (imputed) | 20 (imputed) | 30 | 28 |
| Post-PR | 316 | 127 (real) | 189 (real) | NA | NA |
| Total | 410 | 143 | 209 | 30 | 28 |

Notes: The imputed PR and non-PR approvals were constructed by propensity score matching. The unmatched drugs were excluded in DID models. PR, China’s priority review. NA, not applicable.

**Table S4. Balancing statistics.**

| Variable | Mean(SD) |  |  |  | Mean(SD) |  |  |
| --- | --- | --- | --- | --- | --- | --- | --- |
|  | Real PR | Imputed PR | P value |  | Real non-PR | Imputed non-PR | P value |
| FDA priority review (1/0) | 0.799  (0.402) | 0.831  (0.362) | 0.428 |  | 0.457  (0.500) | 0.500  (0.506) | 0.616 |
| FDA orphan designation (1/0) | 0.540  (0.500) | 0.503  (0.463) | 0.472 |  | 0.315  (0.466) | 0.304  (0.465) | 0.895 |
| FDA accelerated approval (1/0) | 0.206  (0.406) | 0.200  (0.404) | 0.922 |  | 0.047  (0.213) | 0.108  (0.315) | 0.145 |
| FDA boxed warning at approval (1/0) | 0.275  (0.448) | 0.260  (0 .443) | 0.832 |  | 0.3(0.04) | 0.4(0.07) | 0.370 |
| FDA review time, days | 278.7 (279.0) | 353.2 (279.2) | 0.328 |  | 331.5 (212.3) | 364.6 (213.8) | 0.368 |

Notes: PR, priority review; SD, standard deviation.

**Table S5. Characteristics of the matched sample. Values are counts (percentages).**

| Variable | Real +Imputed PR  N=209  (1) | Real +Imputed Non-PR  N=143  (2) | P value  (3) |
| --- | --- | --- | --- |
| Approval class |  |  |  |
| Initial approval | 52(44.4%) | 65(55.6%) |  |
| New indication supplement | 157(66.8%) | 78(33.2%) | <0.01***^a^ |
| Registration class |  |  |  |
| NDA | 131(62.7%) | 87(60.8%) |  |
| BLA | 78(37.3%) | 56(39.2%) | 0.727 ^a^ |
| FDA priority review |  |  |  |
| Yes | 162(77.5%) | 61(42.7%) |  |
| No | 47(22.5%) | 82(57.3%) | <0.01***^a^ |
| FDA orphan designation |  |  |  |
| Yes | 107(51.2%) | 44(30.8%) |  |
| No | 102(48.8%) | 99(68.2%) | <0.01***^a^ |
| FDA accelerated approval |  |  |  |
| Yes | 46(22.0%) | 8(5.6%) |  |
| No | 163(78.0%) | 135(94.4%) | <0.01***^a^ |
| FDA boxed warning at approval |  |  |  |
| Yes | 53(25.4%) | 50(35.0%) |  |
| No | 156(74.6%) | 93(65.0%) | 0.052* ^a^ |
| Pivotal trial type |  |  |  |
| Domestical trial | 61(29.2%) | 58(40.5%) |  |
| Overseas trial | 69(33.0%) | 35(24.5%) |  |
| Global trial enrolling China-based sites | 79(37.8%) | 50(35.0%) | 0.063* ^a^ |
| Therapeutic area |  |  |  |
| Cancers | 97(46.4%) | 52(36.4%) |  |
| HIV/HCV | 20(9.6%) | 4(2.8%) |  |
| Others | 92(44.0%) | 87(60.8%) | 0.002***^a^ |
| FDA review time, days, mean(SD) | 296.12(20.53) | 352.69(18.96) | 0.05* ^b^ |
| Launch delay, days, mean(SD) | 1472.44(96.27) | 2162.11(147.44) | <0.01*** ^b^ |
| Submission delay, days, mean(SD) | 1350.40(98.02) | 1983.94(146.19) | <0.01*** ^b^ |
| NMPA review times, days, mean(SD) | 418.17(13.37) | 527.66(23.80) | <0.01*** ^b^ |

Notes: Dual-matched approvals were not included. ^a^ P values were from chi-square test. ^b^ P values were from independent t-test. PR, priority review. NDA, new drug application. BLA, biologics license application. NMPA, National Medical Product Administration. SD, standard deviation. *p < 0·10 **p < 0·05 ***p < 0·01.

Rosenbaum bounds of the matching for real PR were listed in Table S6. Gamma=1 indicated the usual randomization inference. The gamma denoted the size of potential hidden bias, and larger gamma indicated greater hidden bias. E.g., gamma=2 meant individuals in one group had twice more likelihood to receive treatment than the other. For PR, the P-values were small when gamma=1 or 2, which indicated that, the difference in drug delays between the real PR and its matched pre-PR counterpart were attributed to whether they were approved after the PR was created. However, when gamma became quite large ($\geq$3), the range included both small and large, significant and insignificant, P-values, which indicated the null hypothesis was not rejected. This suggested that when large departure from randomization was present, e.g., one approval had 3/4 or higher chance to be listed in post-PR period while the other only had a chance of 1/4 or lower, the variations in launch delay would be attributed to the hidden bias rather than the policy. Hence, the matching for PR approvals was sensitive to moderate hidden bias.

Rosenbaum bounds of the matching results for real non-PR were not showed because they were unable to examine the potential hidden bias. This was due to that the null hypothesis underlying Rosenbaum bounds was no treatment effect; however, both the real non-PR and its matched pre-PR counterpart were used as control groups, which were not supposed to be affected by the treatment (the PR program). In this case, given that the differences in delays between real non-PR and imputed non-PR were indeed not significant (Table S10), the null hypothesis would never be rejected, resulting in no clear evidence about hidden bias. Taking the matching for real PR as reference, the hidden bias in matching real non-PR might also be moderate.

**Table S6. Sensitivity analysis for the one-tailed P-value for testing the null hypothesis of no treatment effect of treatment periods on drug delays length in post-PR approvals and pre-PR approvals matched for the FDA approval characteristics** **^a^.**

| Gamma | Matching for PR | | | | | | | |
| --- | --- | --- | --- | --- | --- | --- | --- | --- |
|  | Launch delay | |  | Submission delay | |  | Review times | |
|  | $P_{max}$ | $P_{min}$ |  | $P_{max}$ | $P_{min}$ |  | $P_{max}$ | $P_{min}$ |
| 1 | $3.4\times{10}^{-11}$ | $3.4\times{10}^{-11}$ |  | $6.3\times{10}^{-9}$ | $6.3\times{10}^{-9}$ |  | $3.7\times{10}^{-13}$ | $3.7\times{10}^{-13}$ |
| 2 | <${10}^{-15}$ | 0.003 |  | <${10}^{-15}$ | 0.015 |  | <${10}^{-15}$ | 0.003 |
| 3 | <${10}^{-15}$ | 0.258 |  | <${10}^{-15}$ | 0.398 |  | <${10}^{-15}$ | 0.080 |
| 4 | <${10}^{-15}$ | 0.784 |  | <${10}^{-15}$ | 0.862 |  | <${10}^{-15}$ | 0.490 |

Notes: ^a^ the characteristics used for matching were the FDA priority review, the FDA orphan designation, the FDA accelerated approval, the FDA boxed warning at approval, and FDA review time.

**Figure S1.** **Robustness for the impact on launch delay:** **1000 regressions with random assignment of the dual-matched approvals.**

Notes: Most estimations (73.2%) were significant. Average reduction of launch delay was 949.84 days (SD, 233.42).

**Figure S2. Robustness for the impact on NMPA review times: 1000 regressions with random assignment of the dual-matched approvals.**

Notes: Only 18.5% of the estimations were significant. Average reduction of review times was 141.77 days (SD, 58.59).

**Figure S3. Robustness for the impact on submission delay: 1000 regressions with random assignment of the dual-matched approvals.**

Notes: Most estimations (66.3%) were significant. Average reduction of submission delay was 842.38 days (SD 211.54).

Results of the event studies were shown in Figure S4-S6. The main results with the exclusively matched approvals were in Figure S4, and Figure S5 and S6 were sensitivity analysis including the dual-matched approvals. For the period that the PR had not been introduced yet (2007-2014), all the DID estimations were not significant, revealing evidence in favor of the parallel trend assumption holding. The PR’s effects on launch delay and submission delay were significant after its implementation (2016-2018), and gradually reduced thereafter (2019-2023), as shown in Figure S4. If the dual-matched approvals were included as non-PR ones, the PR’s effects were more significant (Figure S5). However, when the dual-matched approvals were included as PR ones, the PR’s effects were not statistically detectable (Figure S6).

**Figure S4. Drug delays event studies: the exclusively matched PR and non-PR approvals were used as the pre-PR samples.**

Notes: (A) launch delay. (B) submission delay. (C) review times. The red dot line denoted 2016, which was the real timing of the implementation of the PR.

**Figure S5. Drug delays event studies: the exclusively matched PR approvals were used as imputed PR approvals, while the exclusively approvals matched non-PR and the dual-matched approvals were used as imputed non-PR approvals.**

Notes: (A) launch delay. (B) submission delay. (C) review times. The red dot line denoted 2016, which was the real timing of the implementation of the PR.

**Figure S6. Drug delays event studies: the exclusively matched PR approvals and the dual-matched approvals were used as imputed PR approvals, while the exclusively matched non-PR approvals were used as imputed non-PR approvals.**

Notes: (A) launch delay. (B) submission delay. (C) review times. The red dot line denoted 2016, which was the real timing of the implementation of the PR.

**Figure S7. Trends of drug delays in the post-PR period.**

Notes: launch delay and submission of the PR approvals were steady during 2017-2022, while delays of the non-PR approvals notably reduced after 2018. Data in 2016 were not shown due to the low sample size. PR, priority review.

**Figure S8. Robustness for the impact on submission delay for orphan drugs: 1000 regressions with random assignment of the dual-matched approvals.**

Notes: Most estimations (68.2%) were significant. Average reduction of submission delay was 1624.85 days (SD, 348.96).

**Figure S9. Robustness for the impact on submission delay for non-orphan drugs: 1000 random assignments of the dual-matched approvals.**

Notes: Only 2.4% of the estimations were significant. Average reduction of submission delay was 337.75 days (SD, 284.10).

**Table S7. Mediation analysis results.**

|  | Mean (robust standard error) |
| --- | --- |
| PR effect on conditional approval ($\beta_{a_{1}}$) | 0.2(0.04)*** |
| Relation between submission delay and conditional approval ($\beta_{b_{1}}$) | -932.4(246.6)*** |
| PR effect on trial type ($\beta_{a_{2}}$) | 0.4(0.1)*** |
| Relation between submission delay and trial type ($\beta_{b_{2}}$) | -836.8(84.8)*** |
| Direct effect ($\beta_{c^{'}}$) | -361.4(178.4)** |
| Indirect effect ($\beta_{a_{1}}\beta_{b_{1}}+\beta_{a_{2}}\beta_{b_{2}}$) | -487.0[-570.8, -412.3] |
| Total effect ($\beta_{a_{1}}\beta_{b_{1}}+\beta_{a_{2}}\beta_{b_{2}}+\beta_{c^{'}}$) | -848.4[-985.1, -712.2] |
| Mediation proportion (Indirect/Total) | 57.5%[48.9%, 67.6%] |

Notes: The sample size was 311, including all the drugs with information on the conditional approval designation. The 95% confidence intervals were in square brackets (built with bootstrapped standard errors, 1000 replications).

**Table S8. Results of Mahalanobis matching.**

|  | Total | Non-PR | PR | Dual-matched | Unmatched |
| --- | --- | --- | --- | --- | --- |
| Pre-PR | 94 | 19 (imputed) | 14 (imputed) | 29 | 32 |
| Post-PR | 316 | 127 (real) | 189 (real) | NA | NA |
| Total | 410 | 146 | 203 | 29 | 32 |

Notes: The imputed PR and non-PR approvals were constructed by propensity score matching. The unmatched drugs were excluded in the DID models in Table S9. PR, China’s priority review. NA, not applicable.

**Table S9. Impacts of the PR on drug delays with Mahalanobis matching.**

| Variable | Launch delay | | Review times | | Submission delay | |
| --- | --- | --- | --- | --- | --- | --- |
|  | (1) | (2) | (3) | (4) | (5) | (6) |
| PR | 178.70 (529.19) | 573.34 (525.83) | 47.37 (139.48) | 124.10 (146.04) | 1.14 (535.25) | 525.41 (515.68) |
| PR$\times$Post-PR | -958.55* (560.26) | -1251.56** (548.77) | -164.99 (141.98) | -230.40 (148.28) | -712.61 (566.70) | -1087.03** (538.21) |
| Constant | 2099.95*** (362.47) | 13312.23 (91010.99) | 747.63*** (84.16) | 32945.56* (17265.86) | 1872.79*** (336.36) | 32383.99 (85997.04) |
| Controls | N | Y | N | Y | N | Y |
| N | 349 | 349 | 349 | 349 | 349 | 349 |
| Adj-$R^{2}$ | 0.0626 | 0.2716 | 0.1876 | 0.3350 | 0.0479 | 0.2697 |

Notes: Observations incorporated the post-PR approvals and the pre-PR approvals that were exclusively matched to either the real PR or the real non-PR. Robust standard errors were in parenthesis. Controls included were the FDA’s designations of priority review, accelerated approval and orphan drug, the FDA’s boxed warning at approval, the FDA review extension, the therapeutic areas [cancers, major infectious diseases (HIV/HCV), or others], the type of the pivotal trial enabling the NMPA approval (domestic trial, overseas trial, or global trial with sites in China), the approval class (NDA or BLA), the marketing class (initial marketing approval or new indication supplement), and the year of the NMPA approval. PR, priority review. NMPA, National Medical Product Administration. NDA, new drug application. BLA, biologics license application. *p < 0.10 **p < 0.05 ***p < 0.01.

**Table S10. Effects of implementing PR among non-PR approvals.**

| Variable | Launch delay | |  | Submission delay | |  | Review times | |
| --- | --- | --- | --- | --- | --- | --- | --- | --- |
| Post-PR | 1031.60 (930.53) | 1222.40* (695.92) |  | 881.01 (840.92) | 1049.72 (642.83) |  | 110.94 (197.59) | 153.56 (150.35) |
| Controls | Y | Y |  | Y | Y |  | Y | Y |
| Imputed non-PR  (exclusively  matched) | Y | Y |  | Y | Y |  | Y | Y |
| Imputed non-PR  (dually  matched) | N | Y |  | N | Y |  | N | Y |
| N | 143 | 173 |  | 143 | 173 |  | 143 | 173 |
| $R^{2}$ | 0.3377 | 0.2891 |  | 0.3716 | 0.3257 |  | 0.3901 | 0.3175 |

Notes: Robust standard errors were in brackets. All the estimations were controlled for the FDA’s designations of priority review, accelerated approval and orphan drug, the FDA’s boxed warning at approval, the FDA review extension, the therapeutic areas [cancers, major infectious diseases (HIV/HCV), or others], the type of the pivotal trial enabling the NMPA approval (domestic trial, overseas trial, or global trial with sites in China), the approval class (NDA or BLA), the marketing class (initial marketing approval or new indication supplement), and the year of the NMPA approval. PR, priority review. NMPA, National Medical Product Administration. NDA, new drug application. BLA, biologics license application. *p < 0.10 **p < 0.05 ***p < 0.01.

**Table S11. Effects of registration class on drug delays**

| Variable | Launch delay | Submission delay | Review times |
| --- | --- | --- | --- |
| PR (vs. non-PR) | -711.06***(167.33) | -594.41***(163.05) | -113.27***(25.85) |
| Supplement (vs. initial approval) | -364.01**(155.86) | -252.10(152.99) | -109.16***(23.32) |
| *Interaction* |  |  |  |
| Non-PR$\times$initial | Reference | Reference | Reference |
| PR$\times$initial | -707.78**(233.70) | -550.01**(226.72) | -152.41**(37.01) |
| Non-PR$\times$supplement | -359.51(276.80) | -191.17 (271.46) | -162.88**(40.09) |
| PR$\times$supplement | -8.36(314.83) | -113.10 (309.67) | 99.70**(45.25) |

Notes: Robust standard errors were in brackets. All the estimations were controlled for the FDA’s designations of priority review, accelerated approval and orphan drug, the FDA’s boxed warning at approval, the FDA review extension, the therapeutic areas [cancers, major infectious diseases (HIV/HCV), or others], the type of the pivotal trial enabling the NMPA approval (domestic trial, overseas trial, or global trial with sites in China), the approval class (NDA or BLA), and the year of the NMPA approval. PR, priority review. NMPA, National Medical Product Administration. NDA, new drug application. BLA, biologics license application. *p < 0.10 **p < 0.05 ***p < 0.01.

1. *JCO Oncol Pract*. 2022;18(9):e1522-e1532. doi:10.1200/OP.21.00909 [↑](#footnote-ref-1)
2. *JAMA*. 2017;317(18):1854-1863. doi:10.1001/jama.2017.5150 [↑](#footnote-ref-2)
3. *Journal of Health Economics*. 2008;27(2):175-200. doi:10.1016/j.jhealeco.2007.10.007 [↑](#footnote-ref-3)
